# An unusual cause of upper gastrointestinal bleeding

**Published:** 2014

**Authors:** Shabnam Shahrokh, Mohammad Reza Zali

**Affiliations:** Gastroenterology and Liver Disease Research Center, Shahid Beheshti University of Medical Sciences, Tehran, Iran

## Question

A 48-year-old man presented to our emergency department with hematemesis and abdominal pain. He was a former heroin addict and had been well until 8 months earlier, when he had epigastric and periumbilical pain, anorexia, 12 kg weight loss and lower back pain.

On Examination, his oral temperature was 36.8˙C, his pulse was regular with a rate of 92 bpm and blood pressure was 160/95 mmhg. He was appeared pale, the rest of physical examinations were unremarkable, except for mild tenderness in periumbilical area.

**Picture A:**
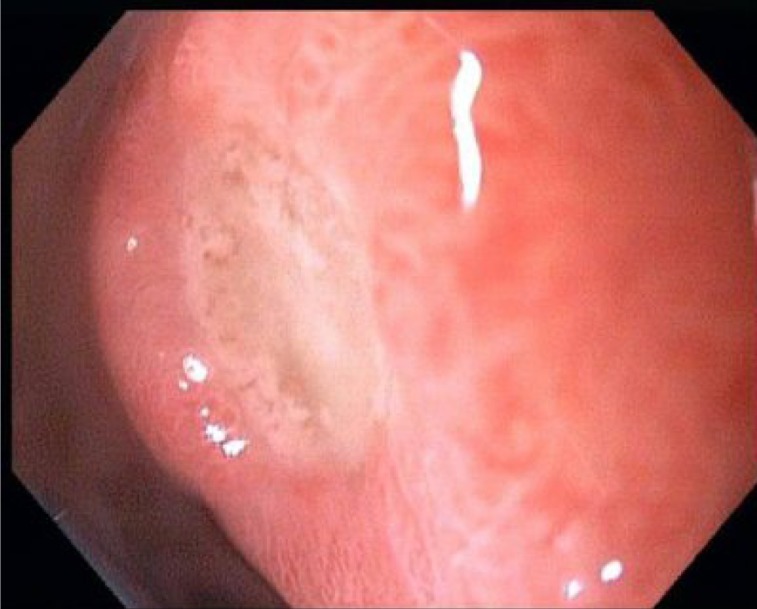


After initial resuscitation, the initial work up included a normal chest radiograph and electrocardiogram (ECG). Laboratory testing on admission revealed a hemoglobin level of 8.3 g/dl (the patient’s last hemoglobin test, which was performed 2 months ago, was 13.2 g/dl). His platelet count is measured at 310×103/ μL, prothrombin time was 1.3 seconds, liver enzymes were within normal limits, ESR was 65 mm/hr and CRP was 43 mg/L. Endoscopy (picture A) revealed a small clean base ulcer in anterior portion of the bulb without stigmata of recent bleeding, otherwise was normal up to second part of duodenum. Because of significant weight loss and abdominal pain, contrast-enhanced computed tomography of the abdomen and pelvic was performed (Picture B).

**Picture B:**
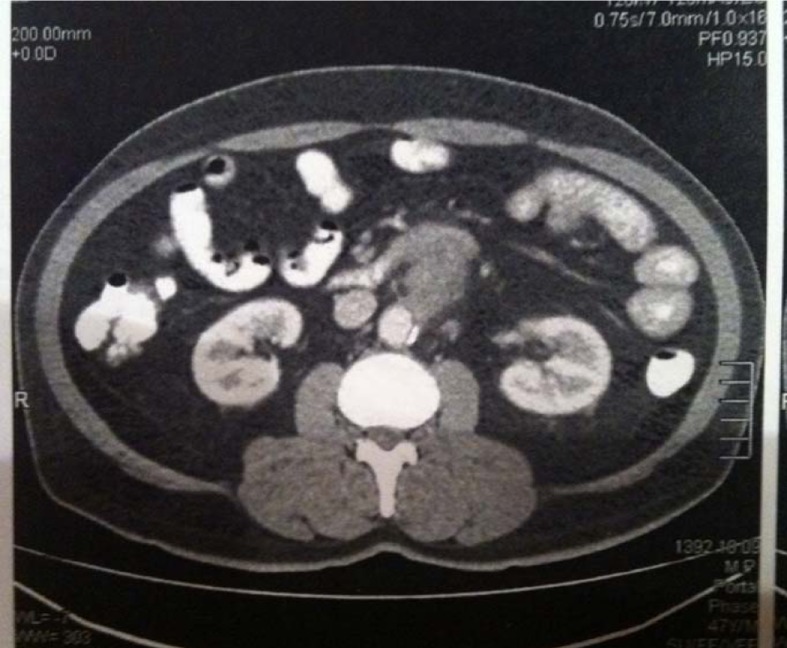


**What is the diagnosis?**

**What is the next step?**

Computed tomography showed a well-defined, amorphous hypodense mass, which had an adhesion from one side to duodenum wall and from the other side to aortic wall. 

With suspicious to aortic aneurysm, CT-angiography was performed, which is shown in the picture below:

**Figure:**
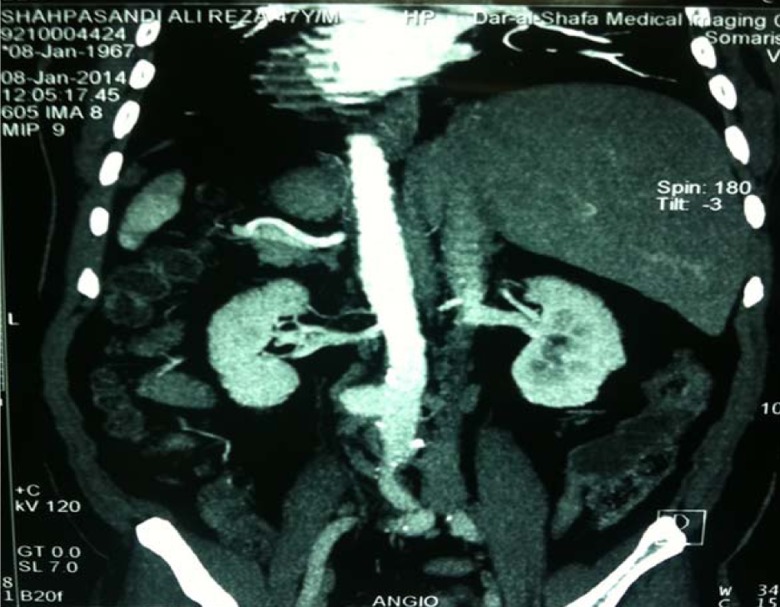


CT-angio revealed two aneurysms on descending thoracic aorta, abdominal aorta and Aortoentric fistula between abdominal aneurysm and duodenum. 

Patient was referred to surgical team and he undergone an emergency excision of saccular infrarenal aneurysm with aortic patch angioplasty and repair of duodenal fistula. TB and syphilis were ruled out. The patient recovered without complication.

## Discussion

Aortoenteric fistula (AEF) a direct communication between the aorta and the gastrointestinal tract is an uncommon cause for acute upper gastrointestinal bleeding with high mortality rate if left untreated. AEF can either be primary, arising from the atherosclerotic aortic aneurysm or uncommon such as infectious aortitis due to syphilis or tuberculosis or secondary (common type), from previous aortic grafting or other secondary causes include penetrating ulcers, tumor invasion, trauma, radiation therapy, and foreign body perforation.

Primary aortoduodenal fistula (ADF) is a rare clinical entity that usually presents with gastrointestinal bleeding that can be occult, intermittent, or massive. The classic triad of massive gastrointestinal bleeding, a pulsatile abdominal mass, and abdominal (or back) pain is seen in small percentage of patients. Repetitive gastrointestinal bleeding heralded most cases.

Other signs and symptoms may include abdominal or back pain, fever, and sepsis.

The third or fourth portion of the duodenum is the most common site for aortoenteric fistulas, followed by the jejunum and ileum.

 A high index of suspicion is needed to establish the diagnosis of an aortoenteric fistula and should be considered in all patients with massive or repetitive upper gastrointestinal bleeding and a history of a thoracic or abdominal aortic aneurysm or a prosthetic vascular graft.

Endoscopy is important primarily to exclude other causes of acute upper gastrointestinal bleeding, such as ulcer disease. Additionally, endoscopy with an enteroscope or side-viewing endoscope may reveal a graft, an ulcer or erosion at the adherent clot, or an extrinsic pulsatile mass in the distal duodenum or esophagus. The best diagnostic test is abdominal computed tomographic scanning, which often shows an aortic phlegmon in the area of the third or fourth portion of the duodenum. Aortography is typically unreliable and not recommended. Exploratory laparotomy is indicated for patients with suspected aortoenteric fistula and severe ongoing bleeding.

 Surgical repair of the aortic aneurysm and fistula is the standard treatment regardless of the cause. Therapy of an aortoenteric fistula due to an infected graft consists of intravenous antibiotics and emergency surgery.
